# Playing TAG with a Bacterial Stress Response

**DOI:** 10.1371/journal.pbio.1001068

**Published:** 2011-05-24

**Authors:** Caitlin Sedwick

**Affiliations:** Freelance Science Writer, San Diego, California, United States of America

**Figure pbio-1001068-g001:**
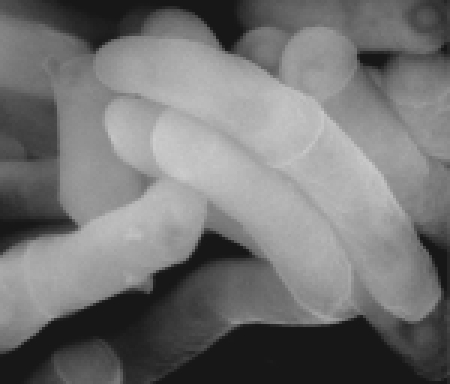
***Mycobacterium tuberculosis*** **adapts to stress by assuming a quiescent state in which it is indifferent to antibiotics. Inhibiting this response sensitizes the bacterium to antibiotic treatment and accelerates drug therapy.** Image credit: Jennifer Griffin, University of Massachusetts Medical School.

[Fig pbio-1001068-g001]Tuberculosis is an age-old scourge of mankind. This chronic disease is caused by a bacterium, *Mycobacterium tuberculosis*, which sets up an initial infection in its victims' lungs. Although the infection can spread to other organs, its most common symptoms are a painful and debilitating cough combined with difficulty breathing. Tuberculosis remains a major public health threat because even the best antibiotics currently available take a long time to clear the infection, and noncompliance with protracted antibiotic regimens can lead to the evolution and spread of drug-resistant strains.

In order to better treat the disease, it will be important to understand how *M. tuberculosis* resists even the most powerful antibiotics for so long. One clue is that most antibiotics work best on rapidly growing populations of bacteria, but it's been observed that once *M. tuberculosis* has established its initial infection, it is capable of dramatically slowing its metabolism and rate of cell division. This has led to the suggestion that the bacterium's retreat into a hibernation-like state underlies its remarkable resistance to antibiotic therapy. How does the bacterium execute this strategy? That's the question that Seung-Hun Baek, Alice Li, and Christopher Sassetti tackled in work published in this issue of *PLoS Biology*.

In order to identify pathways that regulate bacterial growth, Sassetti's group subjected the bacteria to mutagenesis, hoping to disrupt genes that govern growth. They then examined the mutated cells to find ones that failed to arrest their growth in vitro in the presence of low oxygen, which normally causes growth arrest. Next, they used a method called transposon site hybridization to identify all of the mutations that give rise to this altered phenotype. Using this technique, Baek et al. observed that proteins involved in the production of the lipid triacylglycerol (TAG), in particular a protein called Tgs1, were required if bacterial cells were to arrest their growth in a low-oxygen environment.

Tgs1 synthesizes TAG from a precursor molecule called acetyl CoA. But in addition to its role in TAG synthesis, acetyl CoA is also a major substrate of the citric acid cycle, the central metabolic pathway that fuels growth. Sassetti's group therefore wondered whether, under conditions of stress, the bacteria use TAG synthesis to deliberately divert acetyl CoA away from growth-promoting metabolic pathways. Indeed, they found that adding a downstream metabolite of the citric acid cycle prevents bacteria from entering growth arrest after exposure to low oxygen or other stressors (for example, iron deprivation and low pH). Likewise, increasing the level of the citric acid cycle enzyme that competes with Tgs1 for access to acetyl CoA also short-circuits growth arrest. Consistent with the idea that bacteria are diverting acetyl CoA away from the citric acid cycle, the authors' data also show that when Tgs1 is present, more acetyl CoA ends up incorporated into triacylglycerides than is used for energy metabolism.

Because bacteria lacking Tgs1 grow more rapidly than wild-type bacteria, the authors suspected that Tgs1-deficient bacteria might also be more susceptible to antibiotics. To test this idea, they compared the survival of antibiotic-treated wild-type and Tgs1-deficient bacteria in low-oxygen conditions in vitro, and in infected mice. In fact, several different antibiotics killed wild-type bacteria much more slowly than they killed Tgs1-deficient bacteria, indicating that bacterial ability to slow growth through TAG synthesis is an important determinant of antibiotic resistance.

Together, these findings help to illuminate a major pathway that *M. tuberculosis* uses to alter its metabolism in response to environmental stress. This response, argues, could help *M. tuberculosis* prevent overutilization of necessary nutrients, allowing the bacteria to continue living in a hostile host environment. It also, incidentally, helps clarify the contribution of metabolic rate (and TAG synthesis) to the bacteria's ability to resist the best antibiotic weapons humans have yet been able to marshal against them. Now that this relationship is better understood, it may be possible to design a way to deliberately disrupt the bacterial stress response and so improve antibiotic efficacy.

**Baek S-H, Li AH, Sassetti CM (2011) Metabolic Regulation of Mycobacterial Growth and Antibiotic Sensitivity. doi:10.1371/journal.pbio.1001065**

